# Experiences of women enrolled in a prevention of mother to child transmission of human immunodeficiency virus infection programme in Zimbabwe

**DOI:** 10.4102/hsag.v24i0.1088

**Published:** 2019-02-27

**Authors:** Oslinah B. Tagutanazvo, Anna G.W. Nolte, Annie Temane

**Affiliations:** 1 Department of Midwifery Science, University of Eswatini, Kingdom of Eswatini; 2 Department of Nursing, Faculty of Health Sciences, University of Johannesburg, South Africa

## Abstract

**Background:**

Prevention of mother-to-child transmission (PMTCT) programmes have been reported to reduce the rate of transmission of human immunodeficiency virus (HIV) infection by 30% – 40% during pregnancy and childbirth. The PMTCT transmission is achieved by offering HIV prophylaxis or initiating antiretrovirals to pregnant women who test HIV positive. Being aware of the experiences of these women will assist in planning and implementing the relevant care and support. The study was conducted in three phases.

**Aim:**

This article will address phase 1 which is to explore and describe the experiences of pregnant women living with HIV.

**Setting:**

The study setting was a PMTCT site in a Provincial Hospital, in Zimbabwe.

**Methods:**

The study design was qualitative, exploratory, descriptive and contextual. In-depth face-to-face interviews were conducted from a purposive sample of 20 pregnant women. Thematic data analysis was performed.

**Results:**

Six themes emerged: realities of disclosure, a need for quality of life, perceived stigmatisation, inadequate knowledge on infant feeding, continuity of care, empowerment and support.

**Conclusions:**

The study concluded that pregnant women living with HIV require empowerment and support to live positively with HIV.

## Introduction and background

Zimbabwe has an estimated 1.4 million people living with human immunodeficiency virus (HIV), of whom 77 000 are children aged below 15 years who contracted HIV through vertical transmission (UNAID 2015). The HIV prevalence among women and men aged 15–49 years is reported to be about 14% (UNAID 2015). Human immunodeficiency virus responses in Zimbabwe constitute a national emergency, and prevention of HIV infection in infants is an important priority (ICF 2012; MOH & CW 2007; ZIMSTAT 2012). The Zimbabwean government has shown its commitment to the fight against HIV and AIDS through the implementation of the 1999 HIV and AIDS policy, the Zimbabwe National HIV and AIDS Strategic Plan 2006–2010, provision of prevention of mother to child transmission (PMTCT) services and its second edition of the PMTCT policy of 2006 (MOH & CW 2006a). As part of the PMTCT and Paediatric HIV Prevention Treatment and Care National Plan, the latter policy was revised in 2007.

Prevention of mother to child transmission programmes have been reported to reduce the transmission rate of HIV infection by 30% – 40% during pregnancy and childbirth (MOH & CW 2008:i). The Southern African Development Community (SADC), of which Zimbabwe is a member country, acknowledges that the problems related to HIV and AIDS differently impact on men and women, are multi-sectorial and multidimensional, and raise the need to develop effective and relevant policies. In the same report, emphasis is placed on developing programmes that bring about collaboration among partners (SADC Strategy and Programme of Action 2003–2006). This is consistent with Both and Van Roosmalen (2010:1448), who indicate that ‘MTCT programmes miss the opportunity to have an overall positive effect on maternal health because of the way the programmes are structured’.

The PMTCT programme in Zimbabwe focuses on primary prevention of HIV in the general population, prevention of unintended pregnancies among women living with HIV, prevention of HIV transmission from women living with HIV to their infants, treatment as well as care and support for women living with HIV, including their infants and families.

In Zimbabwe, provider-initiated HIV testing and counselling (PI-HTC) is provided for pregnant women presenting at health care facilities, who are not aware of their HIV status. Midwives provide group HIV pre-test counselling to pregnant women during the initial antenatal visit. The women who test positive for HIV receive individual post-test counselling, which is short and focused on PMTCT with emphasis on the need to disclose their status to their partners, relatives or friends for psychosocial support. Their follow-up care and support is not explicit and is partially vested in the hands of friends, lay community counsellors or support groups (Shetty et al. 2008). However, following disclosure, these women need support and skills to deal with various challenges such as the HIV-related stigma, the unpredicted responses of their partners, dealing with issues related to infant feeding and any other matters relating to HIV infection (Nachega et al. 2012). The pregnant women living with HIV have to start on antiretrovirals (ARVs) as prophylaxis or as treatment for their lifetime. Being aware of these women’s experiences will assist in planning and implementing the relevant care and support.

The purpose of this phase of the research was to explore and describe the experiences of pregnant women living with HIV at a Provincial Hospital, who were enrolled in the PMTCT programme. This study is part of a larger study that led to the development of a holistic care model that will be used as a framework by midwives to facilitate quality of life for pregnant women living with HIV.

The study was conducted in three phases addressing the following objectives: *Phase 1*: To explore and describe the experiences of pregnant women living with HIV infection during pregnancy. *Phase 2*: To develop a holistic care model for use by midwives caring for pregnant women living with HIV who are enrolled in a PMTCT programme. *Phase 3*: To evaluate the holistic care model for use by midwives caring for pregnant women living with HIV who are enrolled in a PMTCT programme. This article addresses Phase 1 of the study.

The experiences of the women who were under study and the roles and responsibilities of the midwife as enshrined in the definition of the ‘midwife’ (International Confederation of Midwives [ICM] 2011) were used to identify the relevant care and support the midwives provide. The midwife’s roles and responsibilities include those of carer, counsellor, collaborator and coordinator of care. The midwife works as a coordinator and collaborator of midwifery care in a multidisciplinary team involving obstetricians, nutritionists or dieticians, social workers, paediatricians and traditional birth attendants, in line with the philosophy and model of midwifery care (ICM 2011).

### Definition of concepts

*Experience*: To do or see (something) or have (something) happen to you; to feel or be affected by (something) (*Merriam Webster Dictionary*).

*Human immunodeficiency virus infection*: By damaging the immune system, HIV interferes with the body’s ability to fight the organisms that cause the infection. Human immunodeficiency virus is a sexually transmitted infection.

*Prevention of mother to child transmission of HIV*: The mother to child transmission (MTCT) of HIV refers to the transmission of HIV from an HIV-positive woman to her child during pregnancy, labour, childbirth or breastfeeding.

*Pregnant woman*: A woman who is in the state of carrying a developing embryo or foetus within the female body.

## Research methods

### Study setting

The study was conducted in Zimbabwe at a Provincial Hospital in the maternal and child health (MCH) department. The PMTCT was incorporated in the MCH programme.

### Research design

The research design used in this phase of the study was a qualitative, exploratory approach, descriptive and contextual in nature, to explore and describe the experiences of pregnant women living with HIV. The research approach was chosen because it allows the researcher to collect information from the person experiencing the phenomenon as the focus of the study was on understanding an experience.

### Population and sampling

The accessible population was pregnant women living with HIV who were enrolled in a PMTCT programme at a Provincial Hospital in Zimbabwe.

The site was purposively selected as the researcher has worked as a midwifery lecturer in this setting. The motivation for the study came as a result of the women within the site’s catchment area confiding in the researcher on the challenges they faced as people living with HIV.

A purposive sample of pregnant women who tested positive for HIV as a result of PI-HTC in the PMTCT programme was used. The inclusion criteria were as follows:

- pregnant women who had known their HIV-positive status during pregnancy and were in the second or third trimester,
- participants who were aged 18 years and older. In Zimbabwe, 18 is the legal age of majority; thus, this age group is viewed as capable of providing informed consent.

The participants were identified by the midwife in charge of the MCH department from a queue of pregnant women who had reported for their scheduled antenatal visits. The identified participants were then each taken to a side room where they were introduced to the researcher. The researcher explained the purpose of the study to each of the participants and highlighted that participation was voluntary. Participants were also free to discontinue the interview at any point without any penalty and also without affecting the services they received. The researcher then obtained an informed verbal and written consent before conducting the interviews.

Because of the sensitive nature of living with HIV and the stigma still attached to HIV, access to participants was at the hospital site, instead of participant homes, to maintain their privacy and confidentiality, and to protect them from stigmatisation by society as a whole.

A total of 20 pregnant women participated in the study. Data saturation determined the sample size. According to Polit and Beck (2014), data saturation entails sampling to the point where no new information is obtained and redundancy is achieved. In this study, no new themes or categories emerged from the in-depth interviews after the 20 participants.

### Data collection

Data were collected by the researcher through in-depth individual face-to-face interviews during 2013. The interviews were tape-recorded and conducted privately in side rooms, away from the other women to ensure privacy, anonymity and confidentiality. The interviews were conducted in Shona (one of the vernacular languages in Zimbabwe) and data were also analysed in Shona before being translated into English.

One central question was asked, namely: ‘How has it been for you since the time you were informed that you are living with HIV infection?’

In this study, each individual in-depth face-to-face interview took about 45 min to 1 h per participant. This includes the time taken to create rapport. Field notes were used by the researcher to support the emerging themes and categories.

### Data analysis

Data from the audiotapes and field notes were transcribed and formed a written record of each of the interviews conducted. The researcher engaged an independent coder who carried out an in-depth analysis of the data, while the researcher also analysed the data. The independent coder is a nurse-midwife who holds a doctoral degree and is well versed in qualitative research with an understanding of both languages (Shona and English).

Tesch’s data analysis method (in Creswell 1994:154–156) was used which includes eight steps described as follows:

*Step 1*: The researcher listened to the tapes (recordings) and obtained a sense of the whole, carefully reading through the transcriptions and jotting down some ideas as they came into the researcher’s mind.

*Step 2*: The researcher picked one interview recording at a time and went through it, asking herself what it was about, and thinking about the underlying meaning. The researcher then wrote down her thoughts in the margins.

*Step 3*: Upon completion of the task, the researcher made a list of topics. The researcher then clustered together similar topics in typed form and arranged these topics in columns under major topics, unit topics and topics left over that had no similarity.

*Step 4*: The researcher used the list of topics and went back to the data, abbreviated the topics as codes and wrote the codes next to the appropriate segments of the texts. The researcher then made a preliminary organising scheme to see whether new categories and codes emerged.

*Step 5*: The researcher identified the most descriptive wording for the topics and turned these into categories. Topics that relate to each other were grouped together, thereby reducing the list of categories. The researcher then identified interrelationships between categories.

*Step 6*: The researcher made a final decision on the categories that were then merged into themes.

*Step 7*: Data belonging to each category were identified and put together, then a preliminary analysis was performed.

*Step 8*: All the existing data were recorded.

### Trustworthiness

Trustworthiness was ensured according to the framework by Lincoln and Guba (1985) (credibility, dependability, confirmability and transferability).

### Ethical considerations

Ethical clearance was provided by the academic ethical committee of a university in South Africa. Permission to carry out the study was obtained from the Acting Medical Superintendent of the Provincial Hospital, while written informed consent was obtained from the participants. Ethical considerations adhered to in the study were the following: freedom to participate in or withdraw from the study, freedom from harm, the right to full disclosure, benefits of the study, the risk/benefit ratio, the right to privacy, anonymity and confidentiality (ethics approval number AEC39/02-2011, Faculty of Health Sciences, University of Johannesburg). Anonymity was also achieved by not linking the names of the participants to the data collected and by using identifying numbers.

## Research findings

Six themes emerged from the data. The themes and categories that emerged from this study are summarised in Table 1.

**TABLE 1:** Themes and categories generated from the study.

Themes	Categories
A need for quality life	Pregnant women living with HIV expressed the following: Acceptance of their own HIV status. A need to postpone sexual activity.
Realities of disclosure	Pregnant women living with HIV reported both positive and negative experiences in dealing with disclosure: Positive experience: Safe disclosure. Hope for better results. Negative experiences: Lack of trust. Fear of the unknown. Mixed spousal or sexual partners’ responses following disclosure.
Perceived stigmatisation	Pregnant women living with HIV expressed fear of stigma and negativity from: In-laws following disclosure. Family, friends and the community. Lack of privacy for provision of HIV services.
Knowledge deficit related to pregnancy and HIV progression in pregnancy	Pregnant women living with HIV expressed a lack of knowledge, including: Fear of transmitting HIV to the infant during breastfeeding. Inadequate knowledge on infant feeding options. How ARVs work in their bodies regarding exclusive breastfeeding.
Knowledge deficit related to continuity of care and support	Pregnant women living with HIV expressed: Inadequate knowledge in relation to how to access ARVs. Inadequate knowledge in relation to care by midwives while in labour. Pregnant women living with HIV also reported the lack of psychosocial or emotional support as a challenge in relation to: Inadequate knowledge of support groups. An inadequate supportive environment.
Empowerment by midwives	Pregnant women living with HIV reported midwives as supportive as: Empowered with knowledge on HIV and available treatment. Giving hope for: An improved quality of life.

HIV, human immunodeficiency virus; ARV, antiretroviral.

### Theme 1: A need for quality of life

The need for a quality life, as reported by participants, was described as the acceptance of their own HIV status attributed to the availability and access to ARV drugs, which contribute to the improvement of an individual’s health. Participants who knew someone currently living with HIV and taking ARVs were likely to have hope for survival and accept their HIV-positive status.

One participant had this to say:

‘A.a.a.h, this did not worry me much because most people now are living with HIV so it is no longer scary to live with HIV.’ (Participant 2, female, 26 years old)

Another participant claimed (with a relaxed face):

‘I have seen some women who are living with HIV that is why I accepted my situation…’ (Participant 6, female, 29 years old)

Another participant echoed:

‘Other people are surviving, other people have been living with HIV for years; I will also survive.’ (Participant 1, female, 30 years old)

Participants reported lifestyle adjustments ranging from engaging in protected intimacy to the postponement of intimacy. A participant said:

‘We were advised by midwives at the clinic to use condoms when indulging in sexual intercourse….’ (Participant 4, female, 25 years old)

Another participant stated:

‘Our life is not the same … these days we rarely indulge in sexual intercourse. I have lost the sexual drive … he does not force me when I say I am tired … because I think he is also afraid.’ (Participant 9, female, 35 years old)

This was echoed by another participant as well:

‘We have not yet had sexual activity … I told him that we should use condoms … he said it’s ok but currently I do not have the sexual drive – I am not interested.’ (Participant 15, female, 25 years old)

A participant said:

‘It took me a long time to accept my status; I imagined that I may deliver a baby who is infected with HIV.’ (Participant 10, female, 28 years old)

### Theme 2: Realities of disclosure

Participants reported both positive and negative experiences following disclosure and these were categorised as safe and unsafe disclosures. On safe disclosure, participants were likely to disclose to their husbands and family members in anticipation of support and confidentiality, and this also included those participants who had realised the benefits of taking ARVs.

One participant said:

‘I disclosed to my mother ….she is very supportive. Now she reminds me about taking the ARVs on time’. (Participant 2, female, 26 years old)

Another participant echoed:

‘I placed my ANC (antenatal care) card on the headboard on his side of the bed; he said oh…. Is that what they said (referring to midwives)…, it does not matter? We will take the medication together.’ (Participant 14, female, 26 years old)

On unsafe disclosure, participants feared for a possible breach of confidentiality by their confidantes in terms of gossip, physical abuse, spousal abandonment and marital breakdown.

One participant said:

‘I will not disclose to others …because people talk too much and this may affect my health…so I will keep it to myself.’ (Participant 12, female, 18 years old)

This was echoed by another participant:

‘I cannot even tell my sister because if 1 day we happen to have misunderstandings; she might hurl insults at me…so it is better to keep it to myself.’ (Participant 19, female, 41 years old)

### Theme 3: Perceived stigmatisation

Participants expressed a fear of stigma and negativity emanating from their in-laws and the community. One participant revealed:

‘I cannot disclose to my in laws…I am afraid of being labelled and stigmatised by my in-laws.’ (Participant 2, female, 26 years old)

Another participant said:

‘I am afraid to tell people because I do not know what the people will say about me when they get to know that I am living with HIV.’ (Participant 12, female, 18 years old)

The participants indicated that the environment did not provide adequate privacy, because all clients collecting ARVs for prophylaxis, women who bring their HIV exposed babies for dry blood testing (DBS) at 6 weeks, and pregnant women who recently tested positive were served in the same room which was crowded and did not allow for privacy.

One participant had this to say:

‘The challenge is that we are all seen in the same room for all services i.e. those who are having blood collected for CD4 count, exposed children who have come for DBS and those collecting medications for prophylaxis for the mother and baby. Masvingo is a very small town; you may be staying in Rujeko or Mucheke Township; so these are the people who will tell others about your HIV positive status.’ (Participant 11, female, 33 years old)

### Theme 4: Knowledge deficit related to pregnancy and human immunodeficiency virus progression in pregnancy

Participants expressed a lack of knowledge in relation to pregnancy and HIV progression in pregnancy, as highlighted by a persistent fear of transmitting HIV to their babies and inadequate knowledge about infant feeding options in relation to HIV and ARVs. One participant stated:

‘The midwives said that we should breastfeed only …. the reason is that the medications which are administered to exposed babies as prophylaxis against HIV are only effective in children feeding on breast milk only … I want to protect my baby from HIV infection.’ (Participant 15, female, 25 years old)

Another participant echoed:

‘I have heard about HIV and AIDS but I do not really understand what it is.’ (Participant 4, female, 25 years old)

Participants had concerns about how ARVs work during breastfeeding. A participant said:

‘… I am going to breast feed my baby because I was informed by the midwives that the ARV prophylaxis only works in babies who are breastfeeding exclusively.’ (Participant 12, female, 18 years old)

### Theme 5: Knowledge deficit related to continuity of care and support

Participants expressed inadequate knowledge about how to access ARVs. One participant claimed:

‘… I am not worried about my status because I am already taking ARVs … but I am not sure of whether I will continue taking ARVs.’ (Participant 17, female, 25 years old)

Another participant agreed:

‘… I do not know where I will get the ARVs to continue with my treatment.’ (Participant 4, female, 25 years old)

Participants also expressed a fear of neglect by midwives when they report to the hospital in labour. One participant said:

‘I am not sure of how I will be treated by midwives when they know that they are conducting a delivery on a patient living with HIV… I am not sure of how I can address this.’ (Participant 11, female, 33 years old)

Another participant responded with a question:

‘Do you think that the midwives will be free to touch me during labour?’ (Participant 12, female, 18 years old)

Participants expressed inadequate knowledge about support groups and an inadequate supportive environment as they reported not being aware of where to find support groups, or having fear of accessing them on their own. Thus, participants did not attend the meetings, abandoned the meetings or stopped attending subsequent support group meetings. One participant had this to say:

‘… I was informed about support groups but I am not sure of how I can gain access to the support groups … The midwife just told me to go on my own.’ (Participant 18, female, 27 years old)

Another participant mentioned:

‘I have been to one support group but on the day I attended the meeting; no one noticed my presence and what was being discussed there did not affect me so I left before the end of the meeting … since then I have not attended any other support group meeting.’ (Participant 19, female, 41 years old)

### Theme 6: Empowerment by midwives

Participants reported midwives being supportive and they were hopeful for an improved quality of life attributed to taking ARVs and their ability to make informed decisions on issues related to living positively with HIV.

One participant declared:

‘If I follow the advice I received from the midwives it will be possible for me to survive.’ (Participant 1, female, 30 years old)

Another participant said:

‘My life is the same following accepting my condition … these days it is not scary because now I know that for the rest of my life I will be taking ARVs … I have seen many people who have been sick; and after taking ARVs they are now looking healthy … you can’t even tell that they are living with HIV.’ (Participant 12, female, 18 years old)

Another participant echoed:

‘You should be happy that a timely diagnosis has been made because you will get help to start taking ARVs early and you will remain healthy and if you are sick you will actually recover well.’ (Participant 20, female, 21 years old)

## Discussion

The study findings indicate that most participants reported acceptance of their HIV status. Their acceptance of their positive HIV status was mainly based on the knowledge that treatment is now available to control opportunistic infections, thereby making a contribution to an improved quality of life. Participants also reported that they had observed some people with advanced disease who responded well to ARVs. As a result, participants who had knowledge of someone living with HIV were likely to accept their status and have hope for survival. Nam et al. (2008) reported that having confidence from relatives, friends as well as clinicians and one’s religious grouping contributes to promoting hope and acceptance of an HIV-positive status, thereby enabling patients to develop a positive therapeutic relationship with their ARVs and make lifestyle changes that promote adherence.

Pregnant women living with HIV reported both positive and negative experiences in dealing with disclosure. Positive disclosure beliefs have been reported as significantly associated with lower stigma, greater self-esteem, lower depressive symptoms and better quality of life (Patel et al. 2011:364). The findings in this study indicated that positive experiences following disclosure ranged from safe disclosure, hope for better results and hope in religion. Alternatively, negative experiences ranged from lack of trust, non-decisiveness to disclose, the ‘waiting game’ or waiting period hoping for a better cluster of differentiation 4 (CD4) count, to mixed spousal and sexual partners’ responses following disclosure. CD4 cells are white blood cells which are destroyed by he HIV hence the need to count them in people infected by HIV as they are likely to drop due to destruction by the HIV virus.

Pregnant women living with HIV expressed a fear of being stigmatised as well as the possibility of breach of confidentiality following disclosure to family, in-laws, friends and the community. Nachega et al. (2012:176) indicate that perceptions of HIV-infected individuals show that people in settings with either a high or low HIV prevalence rate continue to perceive HIV-related stigma. This impacts on their willingness to disclose their HIV status to others and their quality of life. The same authors further report that about three-quarters to a third of people living with HIV experience depression, which is attributed to self-stigmatisation and fear of discrimination.

Mcdonald and Kirkman (2011:580), in a study determining use and non-use of ARVs for PMTCT in Australia, reported that the women who participated in the study revealed that taking ARVs was quite distressing and ‘a daily reminder of a serious, stigmatising illness’. However, these same women found it helpful when they re-framed the treatment as helpful to ensure an HIV-negative baby. This is consistent with Nachega et al. (2012:175), who cited five common HIV- or AIDS-related stigmas affecting respondents, including:

people living with HIV/AIDS do not live a long time, people living with HIV/AIDS should be avoided, people living with HIV/AIDS look different and HIV/AIDS is easily transmitted through normal everyday life.

Roopnaraine et al. (2011:652) state that there is stigma attached to joining a group consisting of people living with HIV infection. In a global survey on HIV-related stigma, isolation and sero-status disclosure, Nachega et al. (2012:176) claim that participants stated that HIV-related stigma was still rife and affects the quality of life for people living with HIV, including their determinants to disclose for fear of discrimination.

Mucheto et al. (2011) indicate that women in the PMTCT programme following HIV counselling and testing reported having not been referred for any form of support after being tested. This is in agreement with Bharat and Mahendra (2007:119), who conveyed that HIV-positive people are still under-utilising care and support services. According to these authors, persons living with HIV and AIDS (PLWHAs) can be more effective counsellors for recently diagnosed individuals to confront sexual and reproductive health issues and reduce HIV- and AIDS-related shame and stigma. This is consistent with Muchtler et al. (2011:79), who indicate that treatment advocacy has been reported to complement medical and other social services to prepare people living with HIV on how to access appropriate health care services.

### Limitations of the study

The study was contextual and the results cannot be generalised. Data were collected through in-depth face-to-face interviews, which were audio-taped. This can contribute to participants providing socially desirable responses. Alternatively, they could also have found the use of a voice recorder as intimidating. Both in-depth interviews and naïve sketches could have been used to generate rich data about the phenomenon under investigation. The researcher addressed the latter by adhering to the ethical principles that were observed.

All interviews were conducted and analysed in vernacular Shona and then translated into English. To minimise loss of information during the translation process, verbatim quotes were used, and both an independent coder and a language person well versed in Shona and English were engaged.

## Conclusions and recommendations

Empowerment by midwives was reported to contribute to behaviour change and hope for a quality life. Participants realised that there was hope for a better quality of life beyond a diagnosis of HIV infection, though most of the participants were still processing information in an effort to promote their health. Some of the participants postponed sexual intercourse or stopped having sexual intercourse, while others used condoms.

Lack of stigmatisation and increasing access to ARVs have contributed to an improved health status in people living with HIV. Participants realised the benefit of ARVs and managed to surpass the stigma attached to HIV, even though some of the participants had a fear of stigmatisation from their relatives, friends and in-laws, which contributed to delayed disclosure.

Fear of the care participants will receive from the midwives during labour was evident among participants. This implies that pregnant women living with HIV should be given relevant information explaining the care they will receive in the maternity unit during the process of childbirth to dispel misconceptions.

The intention of the bigger study was to develop a holistic care model for use by midwives to provide holistic care to pregnant women who become aware of their HIV-positive status during pregnancy as a result of PMTCT. In this study, the researcher defined holistic care as the care that addresses the physical, psychological, sexual and social needs of the pregnant women who test positive for HIV during pregnancy. This implies that as the midwife provides care related to HIV infection in pregnancy, the midwife should identify the physical, psychological, social, mental and sexual needs of the pregnant woman living with HIV and address these needs.

‘Facilitating quality of life through empowerment and support’ was regarded as the main concept of the proposed model for use by midwives to provide holistic care to pregnant women who test positive for HIV during pregnancy. The midwife facilitates the process for the pregnant woman to be constantly engaging her ‘self’ to seek appropriate health care resources and adopt appropriate health-seeking behaviours to keep herself healthy after knowing her HIV-positive status. Such self-care by the pregnant women living with HIV ultimately translates to empowerment. These women are empowered to reflect on self, basing their reflections on the information and resources that are available to them to facilitate the adoption of appropriate health promotion behaviours which will help them to achieve optimum health.
